# A Metapopulation Model to Assess the Capacity of Spread of Meticillin-Resistant *Staphylococcus aureus* ST398 in Humans

**DOI:** 10.1371/journal.pone.0047504

**Published:** 2012-10-24

**Authors:** Thibaud Porphyre, Efstathios Stamatios Giotis, David Hugh Lloyd, Katharina Dorothea Clementine Stärk

**Affiliations:** Veterinary Epidemiology and Public Health Group, Department of Veterinary Clinical Sciences, Royal Veterinary College, Hatfield, United Kingdom; INSERM & Universite Pierre et Marie Curie, France

## Abstract

The emergence of the livestock-associated clone of meticillin-resistant *Staphylococcus aureus* (MRSA) ST398 is a serious public health issue throughout Europe. In The Netherlands a stringent ‘search-and-destroy’ policy has been adopted, keeping low the level of MRSA prevalence. However, reports have recently emerged of transmission events between humans showing no links to livestock, contradicting belief that MRSA ST398 is poorly transmissible in humans. The question regarding the transmissibility of MRSA ST398 in humans therefore remains of great interest. Here, we investigated the capacity of MRSA ST398 to spread into an entirely susceptible human population subject to the effect of a single MRSA-positive commercial pig farm. Using a stochastic, discrete-time metapopulation model, we explored the effect of varying both the probability of persistent carriage and that of acquiring MRSA due to contact with pigs on the transmission dynamics of MRSA ST398 in humans. In particular, we assessed the value and key determinants of the basic reproduction ratio (*R*_0_) for MRSA ST398. Simulations showed that the presence of recurrent exposures with pigs in risky populations allows MRSA ST398 to persist in the metapopulation and transmission events to occur beyond the farming community, even when the probability of persistent carriage is low. We further showed that persistent carriage should occur in less than 10% of the time for MRSA ST398 to conserve epidemiological characteristics similar to what has been previously reported. These results indicate that implementing control policy that only targets human carriers may not be sufficient to control MRSA ST398 in the community if it remains in pigs. We argue that farm-level control measures should be implemented if an eradication programme is to be considered.

## Introduction

The emergence of multiresistant staphylococci and particularly meticillin-resistant *Staphylococcus aureus* (MRSA) has focussed attention on the need for better understanding the epidemiology and pathogenesis of staphylococcal diseases [1]. Both hospital- and community-acquired MRSA strains are significant risks for public health, with a mortality rate now surpassing that of HIV/AIDS in the United States of America [2]. Despite the implementation of drastic and costly intervention measures to control infections, the spread in humans remains difficult to curb as new clones showing broader resistance profiles or more complex epidemiology emerge [1]. Concerns were particularly raised with the discovery of livestock-associated MRSA (LA-MRSA) strains in humans that may further accelerate the creation of multidrug-resistant bacteria through exchange of resistance genes between human and animal bacterial strains [3].

MRSA sequence type (ST)398 is the most commonly reported LA-MRSA strain [3]. While MRSA ST398 has been mainly associated with pig populations [4], it has also been reported in other animal species such as cattle [5], chicken [6], horses [7], and companion animals (i.e. dogs and cats [8]). To date, this clone has been identified as an occupational health risk for farmers, veterinarians and their families in several European countries [9], and North America [2], [10]. However, the transmission dynamics of MRSA ST398 between at-risk populations, notably to those not directly exposed to live animals, remain unclear.

Transmissibility of MRSA ST398 among hospitalised humans is considered relatively low when compared with other nosocomial strains [11]. However, this is in contrast with the emergence of MRSA ST398 isolates reported from Dutch patients showing no risk factors for acquisition and no reported links to livestock [12]. Although a human-specific *Staphylococcus aureus* ST398 clone has been recently identified [13], the spread of a livestock-specific clone into the community cannot be ruled out. In this paper, we aim to contribute to the understanding of how MRSA ST398 may spread and persist in the community despite such a low transmission probability.

The presence of persistent carriers and the recurrent acquisition of LA-MRSA from pigs have the potential to alter its transmission dynamics. Both affect transmission rates by extending the duration of LA-MRSA carriage, thereby increasing the chance that a carrier would contaminate a healthy individual [14]. Currently, little is known regarding the impact of persistent carriage and recurrent LA-MRSA acquisition from live animal carriers upon the transmission dynamics of MRSA ST398 in humans. However, understanding the transmission dynamics of LA-MRSA carriage into the community from a given source pig farm is essential in designing effective control policies. Here, we investigate the capacity of MRSA ST398 to spread into a hypothetical human population subject to the effect of a single LA-MRSA-positive commercial pig farm. To reach this goal, we developed a metapopulation model that describes how LA-MRSA is transmitted within and between the different at-risk populations. We then explored which of the model’s parameters impacts most on the transmission dynamics for different scenarios of probability of persistent carriage. In addition, we further evaluated, for each tested scenario, the probability for MRSA ST398 to spread into a totally susceptible population, such as the UK, from an imported human case and in the case where MRSA ST398 is present in a commercial pig farm.

## Methods

### The Metapopulation Framework

**Figure 1:**
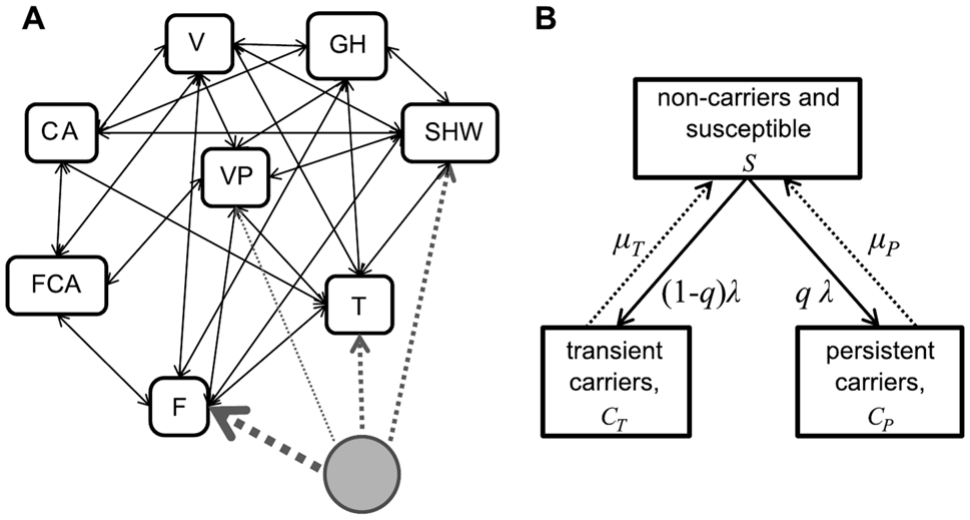
Model structure. (A) Representation of the structure of the metapopulation model for transmission of MRSA ST398 from a single pig farm to the community. The metapopulation comprises eight populations (squares) of various size, species and exposure to the pigs (grey circle) present in a single pig farm. Among these populations, two involve dogs and cats (CA: companion animals, FCA: companion animals in the pig farm). The remaining six populations involve humans with varying level of exposures to LA-MRSA and their families (F: workers of the pig farm, VP: pig veterinarians, T: transporters, SHW: slaughterhouse workers in dirty zone, V: small animal veterinarians, GH: general human population). Solid arrows represent contacts between individuals of different populations. Dotted arrows represent the exposure to pigs, with their width indicating the level of this exposure. (B) Schematic for transmission dynamics of MRSA ST398 between carriage states. Individuals are grouped into three carriage states: non-carriers and those susceptible to be colonised by LA-MRSA, showing either transient or persistent carriage. Straight arrows represent transition between carriage states with solid lines representing transmission and dotted lines representing natural clearance of carriage (see Table 1 for parameters values and definition).

A stochastic, discrete-time metapopulation model was developed to investigate the capacity of MRSA ST398 to spread into a human population subject to the effect of a single LA-MRSA-positive commercial pig farm. With a time step of one day, the model explicitly represents the transmission dynamics of LA-MRSA within and between at-risk populations. Although the model considers all direct transmission pathways involving contacts between humans and companion animals, we ignore transmission through indirect pathways such as via the environment (due to large uncertainties in transmission parameters) and contaminated meat [15]. Figure 1A illustrates how the metapopulation is structured and describes each population in regard to the intensity (in terms of frequency) of contact with the pigs present in the farm of interest.

**Table 1:** Parameters involved in the transmission model for MRSA ST398.

Parameters	Description	Value (unit)	References
*λ_i_*	Proportion of susceptible individuals who acquires MRSA per unit of time		
*β*_0_	Per-contact transmission probability for MRSA non-ST398	0.13	[20]
*ξ*	Relative transmission risk for ST398 MRSA compared to non-ST398	0.17	[11]
*β*	Transmission probability for MRSA ST398	*ξβ*_0_	
*µ_T_*	Clearance rate for transient carrier	0.94 (d^−1^)	[21]
*µ_P_* *	Clearance rate for persistent carrier	0.057 (d^−1^)	[23]
*δ*	Probability of acquiring MRSA ST398 from contact with pigs	*δ*_0_ = 0.1	[5]
*Q*	Probability of being a permanent carrier	Tested values: 0.05,0.1,0.2,0.35	

* Assuming clearance rate for persistent LA-MRSA carriage in humans is equivalent to that in pigs. This value is conservative since MRSA ST398 is adapted to pigs.

The metapopulation is composed of eight populations of finite size, defined by professional activities that may be at risk of acquiring LA-MRSA. Among those, five have direct and indirect contact with pigs. These are: all farm workers (F) and farm companion animals present (FCA) in the pig farm, the veterinary practitioners (VP) monitoring the health status of the farm, the transporters (T) that transport pigs to the abattoir, and all the slaughter house workers (SHW) in the dirty zone of the abattoir where live animals are handled (Figure 1A). We also considered that, despite not being in direct contact with pigs, small animal veterinarians (V) are potentially at-risk of becoming LA-MRSA carriers due to their exposure to a large number of companions animals and humans during their daily consultations [16]. The two remaining populations are the general human (GH) population and companion animals (CA) that do not have direct exposure to pigs.

Each human population comprises those that are directly exposed to pigs and their family.

### MRSA ST398 Transmission Processes

#### Acquisition processes within and between populations

Whilst invasive infections caused by LA-MRSA have been rarely reported, exposure to individuals who carry LA-MRSA in their nostril represents a significant risk for public health. As such, we ignored disease in our model and only considered nasal carriage. Therefore, in each population *i*, individuals pass through two epidemiological states; they are either Susceptible (*S_i_*) or Carrier (*C_i_*). The latter state includes all individuals that carry LA-MRSA and can potentially transmit bacteria to new individuals. We further classified individuals present in the *C_i_* state on the basis of the duration of their nasal carriage as argued by van Belkum et al. [17]: LA-MRSA carriage may be persistent (*C_Pi_*) or not (*C_Ti_*), as illustrated in Figure 1B. For simplicity, we used the word ‘transient’ to describe all non-persistent nasal carriers groups, historically assigned as ‘intermittent’ and ‘noncarriers’. Considering a closed population, the creation of new susceptible individuals is due to clearance of LA-MRSA carriers. Individuals with persistent or transient carriage show distinct clearance rates, recovering from carriage to the susceptible class with constant rates, respectively *µ_P_* and *µ_T_*. In this study, we also assumed that the probability for an individual that newly-acquired LA-MRSA presents a persistent carriage, *q*, is constant throughout time and does not depend on where individuals carry MRSA ST398. The continuous-time analogue of our model of transmission dynamics of MRSA ST398 within a given population *i* can therefore be expressed by the following equations:
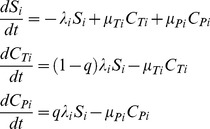
(1)where *i* ∈ {F, VP, …, GH}; and *λ_i_* is the proportion of susceptible individuals of each population *i* that acquires LA-MRSA per unit of time.

As long as individuals carry LA-MRSA in their nostrils, MRSA ST398 is considered to be transmitted directly through physical contact with susceptible individuals or through short-range airborne contamination occurring during conversation. We take LA-MRSA transmission to be frequency-dependent, whereby *λ* in each population *i* is *λ_i_ = βp_i_C_i/_N_i_* where *C_i_* = *C_Ti_* + *C_Pi_* is the total number of LA-MRSA carriers, persistent or transient, in the population *i*; *β* is the transmission probability per contact; *p_i_* is the average number of daily contacts between each members of the population *i*; and *N_i_* is the total number of individuals in the population *i*. Because our model operates in discrete time and involves populations of various sizes, the number of individuals that have newly-acquired LA-MRSA in the *i* ^th^ population, *λ_i_S_i_*, was approximated by a binomial distribution, where the probability that each individual acquires LA-MRSA *m_i_* per time-step is:
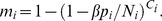
(2)


As within populations, transmission of LA-MRSA between populations is limited to contacts (physical or short-distance air-borne) between members of populations. Also, all contacts within and between populations were assumed to provide the same probability of transmission. We further consider that no carrier and non-carrier individuals move between populations (i.e. they do not change profession) throughout the study period and that transmissibility and clearance rates do not vary between human and animal populations. We therefore defined class *j*, with *j*∈{F,VP,…,GH}, which refers to the populations that are in contact and potentially transmitting LA-MRSA to members of the population *i*. Let us now assume that all members of the *i* ^th^ population would contact, on average, *p_i,j_* members of the *j* ^th^ population per unit of time. In this context, the probability of acquiring LA-MRSA *M*_i_, accounting for the acquisition of LA-MRSA by susceptible individuals of the population *i* due to contacts with members of the population *j,* was calculated in a manner analogous to *m_i_*:
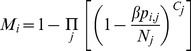
(3)


The numbers of carriers whose carriage is persistent and of those whose carriage status has been cleared at each time step were similarly generated from binomial approximation with probability *q* and *µ*_T_ or *µ*_P_, respectively.

#### Exposure to pigs

Regular exposure to pigs is of special interest when studying the capacity of MRSA ST398 spread since pigs are a source of LA-MRSA in humans [15]. To represent the discontinuous exposure of individuals to pigs that may occur throughout time, a periodic function *Δ_Ti_*(*t*) was constructed such that it equals to *δ* when *t* = {1*Γ_i_*, 2*Γ_i_*, 3*Γ_i_*…} and zero otherwise; where *δ* is the probability that susceptible individuals in each exposed population acquire LA-MRSA due to contact (direct or indirect) with pigs, and 1/*Γ*_i_ is the frequency of contacts individuals from the *i* ^th^ population have with pigs. We assumed that acquiring MRSA ST398 from an external source (i.e. pigs) occurs regularly with a probability *δ*, constant for all exposed populations. The number of individuals with newly-acquired LA-MRSA in the *i* ^th^ population due to contact with members of the *j* ^th^ population was then generated assuming that individuals regularly exposed to pigs were more likely to acquire first LA-MRSA from positive pigs than from other sources.

The value of 1/*Γ*_i_, which measures the extent to which each population is involved in the pig production chain, can be derived from the system and volume of production of the pig farm of interest. For the purpose of this study we considered a typical commercial pig farm using a batch production system, sending every 3 weeks a single lorry of pigs to the abattoir. As a consequence, transporters and slaughterhouse workers involved in this metapopulation are exposed to pigs once every three weeks (*Γ_i = _*21 days), whereas pig farmers have a daily exposure to pigs (*Γ_i = _*1 day). Similarly, we assumed that pig veterinarians visit a given pig farm once every three months (*Γ_i = _*90 days, [18] section H1.2).

### Population Structure and Contact Matrix

#### Population structure

To illustrate how the model performs, we considered a single pig farm in Norfolk, East Anglia, UK, where there is a high density of pigs. Details regarding the population structure of the metapopulation are provided in the electronic supplementary material (Text S1). Briefly, we assumed that people do not acquire LA-MRSA from more than one source farm. The size of the metapopulation is then equivalent to the density of human population per commercial pig farm in Norfolk (i.e., 4984 individuals per pig farm). Given the average household size in the UK is 2.4 (see details in Text S1) and that pig farms in the UK have on average 3.9 farm workers [19], we considered that the population F consisted of 9 individuals. Using similar methodology, we populated all remaining populations of the metapopulation: the size of the populations SHW, T, V and VP finally accounted for 97, 34, 16 and 7 individuals, respectively. We also considered that the study area shows a mean number of companion animals (i.e. dogs and cats) per household similar to national values, with 0.75 dogs or cats present per household. Overall, the metapopulation included 1588 companion animals and 4984 humans; among which 147 had regular contacts with pigs.

#### Contact structure

In the absence of any empirical data measuring amount of contact within and between members of each population in Norfolk, we considered that the number of contacts *p_i,j_* is the product of three processes generating contact: contacts during work activities (A*_i,j_*), contacts between household members (B) and contacts during other daily activities (C*_i,j_*, e.g. leisure, or shopping). Based on this assumption, the contact matrix *P_i,j_* was built such that the diagonal provides the number of contacts within each population as the sum of these three processes, whereas household contacts were excluded for all remaining arrays. Values of A*_i,j_* were computed as a function of the *j* ^th^ population size, the proportions of active individuals (i.e. those that actively participate in the network of contacts between populations, not household members) and *Γ*_i_. We also assumed that the proportion of contacts that emerges from work or in their household are both constant for each population. Values of C*_i,j_* were then derived from the UK subset of the POLYMOD study [20], a cross-sectional population-based survey of individuals in eight European countries in the year 2005–2006. Number of contacts within the GH population was also directly derived from the POLYMOD outcome.

**Figure 2:**
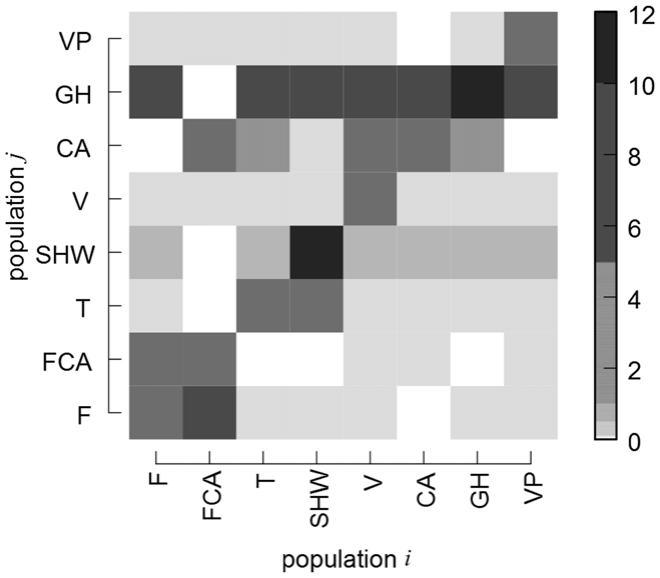
Contact matrix. Contact matrix showing the average daily number of contacts *p_i,j_* between individuals of the metapopulation (see Figure 1 for details).

Figure 2 shows the matrix of the average number of daily contacts occurring between individuals within and between populations. We note that *p_i,j_* ≠ *p_j,i_*. This is because members of two populations may not share similar experience for the same contact event. For example, whilst a pig veterinarian would contact all farm employees in his regular visit to the pig farm, all pig farmers would only have a single contact with the pig veterinarian during his visit. Details regarding the estimation of the contact values are provided in Text S1.

### Parameter Values

The parameters for the model were derived from the published literature on the epidemiology of MRSA non-ST398, MRSA ST398 and *S. aureus*. The values and sources are summarized in Table 1.

Transmission probability, *β*, of MRSA ST398 was directly extrapolated from that of MRSA non-ST398, *β*_0_, such as *β* = *ξβ*_0_. The parameter *ξ* is the relative transmission risk for MRSA ST398 compared to non-ST398 observed in 94 Dutch hospitals [11]. Direct extrapolation was possible based on two assumptions: (1) that both MRSA ST398 and non-ST398 patients involved in the Dutch study show similar contact patterns; and (2) that the relative risk value obtained in Dutch hospital settings is representative to the relative risk occurring in the community. Finally, the probability that a contact with a non-ST398 MRSA carrier would result contamination, *β*_0_, was derived from published experimental results [21], under the hypothesis that interactions likely to result in nasal carriage are, on average, being made once between individual contacts.

Observational studies have shown that most (31 out of 33) newly LA-MRSA-positive field workers after short-term occupational exposure to pigs and veal calves were cleared of LA-MRSA after one day [22]. Under the hypothesis that persistence of MRSA ST398 carriage is related to the intensity of contact with livestock [23], we assumed that all positive samples were from transient carriers and extrapolated the clearance rate for transient carriage such as *µ_T_* = 0.94 d^−1^. No data exists on the duration of persistent carriage in humans. As such, to inform our model, we considered that MRSA ST398 carriage for persistent carriers (1/*µ_P_*) lasts on average 17.4 days [24] as shown in pigs. Although this value is likely to be conservative since MRSA ST398 is adapted to pig, this coincides with the observation that LA-MRSA colonised pig farmers may carry infection for more than 14 days [25].

### Simulation and Analyses

Little is known regarding the probability of persistent carriage *q* for LA-MRSA. In the literature, the proportion of persistent hospital-acquired MRSA carriers ranges from 10% to 20% [23] and, if including *S. aureus* data, may be as high as 35% [26]. Recently, Graveland *et al*. [23] reported that only 5–10% of humans with recurrent contact with animals showed persistent carriage with LA-MRSA. To explore how frequently persistent carriage might occur within a population and evaluate its impact on the transmission dynamics of MRSA ST398, we considered four scenarios of *q* with *q* = 0.05, 0.10, 0.20, and 0.35.

In the case where acquisition of MRSA ST398 from an external source (i.e. pigs) occurs only once among individuals of the metapopulation, we explored the effect of parameter variations on the capacity of LA-MRSA to spread. For each scenario, the capacity of MRSA ST398 to spread and persist in this metapopulation was evaluated using the basic reproduction number, *R*_0_ [14]. This ratio measures the expected number of secondary cases that occur within a totally susceptible population [14]. Empirically, assuming a relatively large susceptible population, a value of *R*_0_>1 indicates that disease will spread whereas a value of <1 indicates that a self-sustaining epidemic is not possible and that LA-MRSA will disappear from the metapopulation [14]. In the context where contacts between individuals are heterogeneous such as in our metapopulation, *R*_0_ can be estimated using the spectral radius of the matrix (the so-called “next generation matrix”) whose elements are the expected number of new cases (either transient or persistent) in each contact population generated by a single carrier (either transient or persistent) present in the metapopulation [27]. However, estimating *R*_0_ may be limited by the level of uncertainty present around parameter values. To account for such effect, the distribution of *R*_0_ estimates for each tested scenario was computed over 20,000 independent randomly generated sets of model parameters. To allow variations around each epidemiological and contact parameters (*n* = 57), the range of variation is taken as identical for all parameters, with perturbation varying uniformly within the space encompassing 25% around their set value. For simplicity, however, a sensitivity analysis (see details below) was carried out to reduce the set of parameters to the most influential ones. Parameters were thus randomly sampled from their uniform distribution if they were found influential in the sensitivity analysis, or fixed to their baseline configuration otherwise.

We define which model parameters influence the most MRSA ST398 *R*_0_ value by computing the total sensitivity index *D_Ti_* using the extension of Fourier amplitude sensitivity test (FAST) as described in Saltelli *et al.* [28]. The extended FAST method is a variance-based, global sensitivity analysis technique that has been largely used for studying complex agricultural, ecological and chemical systems (see [29], [30] for examples). Independently of any assumption about the model structure (such as linearity, monotonicity and additivity of the relationship between input factors and model output), the extended FAST method quantifies the sensitivity of the model output (here *R*_0_) with respect to variations in each input parameter by means of spectral analysis. It provides measures of the amount of variance of *R*_0_ that arise from variations of a given parameter in what is called a total sensitivity index, *D_Ti_*. It therefore captures the overall effect of parameter variations (i.e., including first- and higher-order interactions between model parameters) on *R*_0_ changes. For example, a value of *D_Ti_* = 0.10 indicates that 10% of the total recorded variation of *R*_0_ is explained by the parameter under consideration. To simplify interpretation, the proportion *D_i_*/*D_Ti_* was computed to inform on the fraction of the total effect (*D_Ti_*) attributed to the first-order (direct) effect, *D_i_*. It should be noted that the interest of performing the extended FAST in this study lies not only in revealing to what extent the uncertain parameters affect the model response (here *R*_0_), but also in determining where efforts should be allocated to limit the spread of LA-MRSA in the metapopulation, considering both direct and joint (interactions) effects. Comparison between *D_Ti_* values for each epidemiological and contact parameters (*n* = 57) was based upon the range of variation previously described, that is varying uniformly within the space encompassing 25% around their set value. Based on these analyses, we defined parameters showing the greatest effect on variation of *R*_0_ as those having a *D_Ti_* greater than 0.1.

In addition to the computed mean *R*_0_ value, we further estimated the maximum time required by MRSA ST398 to be eliminated from the metapopulation. This provides information on how long LA-MRSA would naturally remain in the population if a single incursion event occurred. To reach this goal, we followed the evolution of the weekly probability of MRSA ST398 occurrence of a given year. First, we initially seeded the stochastic model described above with one pig farmer that acquired and is permanently carrying LA-MRSA. This was based on the assumption that pig farmers are the most likely population susceptible to first acquire LA-MRSA. Then, for each week after the incursion, we estimated the probability of MRSA ST398 occurrence by computing the proportion of simulations where at least one case remained in the metapopulation.

However, it is most likely that the acquisition of MRSA ST398 by farmers originates from contact with pigs. We explored both changes through time of the probability of LA-MRSA occurrence and of the prevalence of MRSA ST398 carriage in humans when regular exposure to positive pigs occurs. We fixed the probability of acquiring LA-MRSA *δ* equal to *δ*_0_ = 0.1. The relation between probability of acquiring LA-MRSA and prevalence of animals is unknown in the pig farming context. However, the prevalence of farmers with close contact with veal calves is closely related to the percentage of LA-MRSA positive calves on a farm [5]. From this study, individuals working in farms where 50% of animals were positive had 10% chance to carry LA-MRSA, which motivates our choice *δ* = *δ*_0_. The effect of variations in this baseline scenario will be further explored.

Due to the stochastic nature of the model and the strong heterogeneity of contacts, simulation was replicated *(n* = 1300) until we reached a stable average behaviour. All analyses and implementation of the model were conducted in R version 2.12 [31].

## Results

### Influential Parameters

Based on values of the total sensitivity index *D*_Ti_ calculated on *R*_0_ estimates, parameters for transmission probability, *β*, and clearance rate from carriage for persistent carriers, *µ_P_*, showed the greatest impact on the transmission dynamics of MRSA ST398 for all four scenarios (Table 2). Influence (in term of rank and *D*_Ti_ values) was relatively constant throughout the scenario for most parameters. As was expected, the effect of clearance rate from carriage for transient carriers, *µ_T_*, was dependent on the probability of persistent carriage *q*. When *q* increased, the influence of *µ_T_* decreased from accounting for 11.2% of *R*_0_ variations to a mere 0.3%. Although variation of *R*_0_ was mostly sensitive to changes in the epidemiological parameters (i.e. *β*, *µ_P_* and *µ_T_*), with more than 76% of the total recorded variation of *R*_0_, some contacts parameters yielded some influence on *R*_0_. For instance, the frequency of contacts occurring within the SHW (*p*_SHW,SHW_) and the GH populations (*p*_GH,GH_) accounted for about 17 to 22% of the variance of *R*_0_ (Table 2).

**Table 2:** Comparison of variable influence on the basic reproduction number (*R*_0_) for MRSA ST398 in humans between each considered scenario of probability of persistent carriage *q.*

	*q* = 0.05			*q = *0.1			*q* = 0.2			*q = *0.35		
Parameters	Rank	*D_Ti_*	*D*_i_/*D_Ti_* (%)	Rank	*D_Ti_*	*D*_i_/*D_Ti_* (%)	Rank	*D_Ti_*	*D*_i_/*D_Ti_* (%)	Rank	*D_Ti_*	*D*_i_/*D_Ti_* (%)
**All parameters**												
*β*	1	0.526	97.9	1	0.5	97.8	1	0.459	97.4	1	0.429	97.2
*µ_P_*	2	0.126	96.8	2	0.235	97	2	0.335	97	2	0.392	96.9
*p*_SHW,SHW_	3	0.12	84.2	3	0.113	84.1	3	0.103	83.5	3	0.096	83.3
*µ_T_*	4	0.112	96.4	5	0.046	95.7	5	0.013	92.3	5	0.003	100
*p*_GH,GH_	5	0.093	81.7	4	0.087	80.5	4	0.079	79.7	4	0.073	80.8
*P*_GH,SHW_	6	0.001	100	6	0.001	100	6	0.001	100	6	0.001	100
*Total*		0.982	94		0.985	94.1		0.992	94.2		0.995	94.5
**Contact matrix alone**												
*p*_SHW,SHW_	1	0.522	86	1	0.522	86	1	0.522	86	1	0.522	86
*p*_GH,GH_	2	0.466	85	2	0.466	85	2	0.466	85	2	0.466	85
*p*_GH,SHW_	3	0.004	100	3	0.004	100	3	0.004	100	3	0.004	100
*Total*		1.023	83.5		1.023	83.5		1.023	83.5		1.023	83.5

Variable’s influence was measured using global sensitivity analysis.

*D_i_* and *D_Ti_* correspond to the first-order (direct) and total sensitivity indices, respectively.

### Capacity of MRSA ST398 Spread

When comparing the impact of perturbations applied to *β* and the clearance rates *µ_T_* and *µ_P_* on the reproduction number *R*_0_ for the four scenarios considered, perturbations had little impact on *R*_0_. For a probability of persistent carriage *q* = 0.05, values of *R*_0_ constantly remained below 1 and averages at 0.57 (min: 0.34, Max: 0.92). As expected, *R*_0_ increased with *q*. Over the 20,000 iterations, mean *R*_0_ was 0.82 (min: 0.49, Max: 1.31), 1.32 (min: 0.78, Max: 2.10) and 2.07 (min: 1.23, Max: 3.31) for *q* = 0.1, *q* = 0.2 and *q* = 0.35, respectively. However, the impact of perturbations in the influencing parameters remained relatively stable, accounting for 17.9% to 19.8% of the variation around the average estimates.

**Figure 3:**
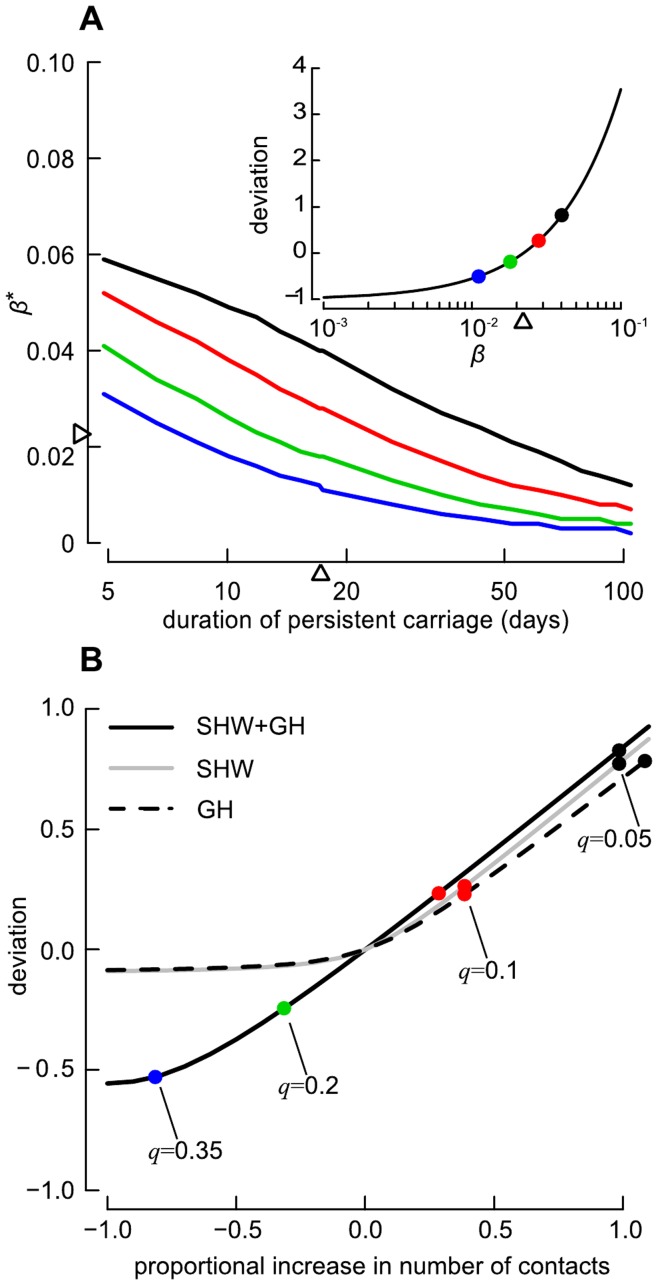
Influence of parameter variations on *R*_0_. (A) Line plot showing the evolution of the critical values of the transmission rate (β*) that would reverse the net reproduction number (*R*_0_) for increasing values of duration of persistent carriage (1/*µ_P_*) and for each considered scenario of the probability of persistent carriage *q*. (B) Line plot showing the deviation in *R*_0_ estimates attributable to changes in the number of contacts within the SHW (*p*_SHW,SHW_) and/or the GH (*p*_GH,GH_) populations. Insert in A shows the deviation in *R*_0_ estimates attributable to changes in *β*. Large dots in the inset of A and in B mark the required changes in parameters value for *R*_0_ = 1 for each considered scenario of the probability of persistent carriage *q*. Triangle dots in A mark baseline values for parameters.

Varying *β*, *µ_P_*, *p*_SHW,SHW_ and *p*_GH,GH_, we seek to define the critical point that would reverse the epidemiological behaviour of LA-MRSA in the metapopulation for all scenarios. Figure 3A shows that: (1) for all scenarios of *q*, there are optimal combinations of *β* and *µ_P_* that allow LA-MRSA to spread in the metapopulation; and (2) with increasing value of *q*, both duration and transmission probability required for *R*_0_>1 would be reduced. For example, considering the situation where the duration of persistent carriage is fixed to its baseline value, the transmission rate critical value (β*) would be 0.04, 0.028, 0.018, 0.011 when *q* = 0.05, 0.1, 0.2 and 0.35, respectively. Conversely, for LA-MRSA to spread with *β* at its baseline value, persistent carriage should last at least 49, 25, 13, and 8 days when *q* = 0.05, 0.1, 0.2 and 0.35, respectively.

In Figure 3B, we examined the influence of changes in the average number of daily contacts with the SHW and GH populations. Increasing the average number of daily contacts in these populations increases the value of *R*_0_. While an increases of 30–40% would allow LA-MRSA to spread in the metapopulation (*R*_0_>1) despite *q* = 0.1, doubling *p*_SHW,SHW_ and/or *p*_GH,GH_ would be required to significantly alter the mean transmission dynamics of LA-MRSA when *q* = 0.05. We further note that, when *q*≥0.2, concentrating efforts on reducing the number of contacts on a single population would not be effective, with deviations from the mean estimate of *R*_0_ not exceeding 10% for both scenarios. Only a simultaneous reduction of 30% to 80% in the number of contacts in these two populations may drive LA-MRSA to fade away when *q*≥0.2.

### Time to Elimination

**Figure 4:**
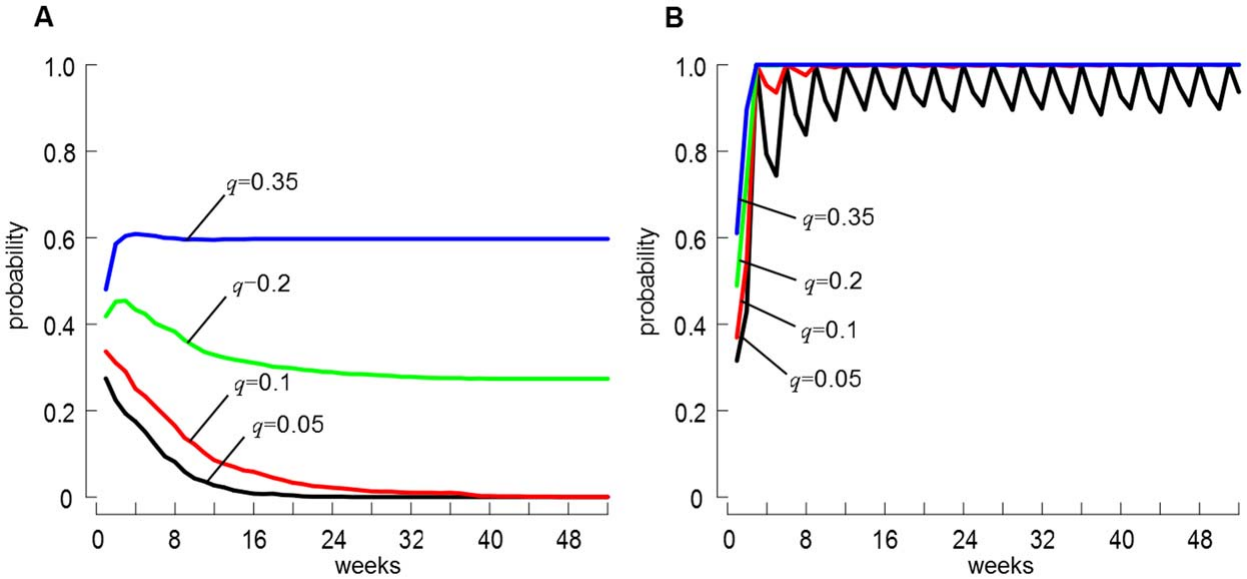
Weekly evolution of the probability of MRSA ST398 occurring in the metapopulation. Weekly evolution of the probability of MRSA ST398 presence in the metapopulation after: (A) a single persistent acquisition in humans working in the pig farm, and (B) multiple incursions in humans due to frequent contacts with pigs. Solid line represents outputs for each considered scenario of the probability of persistent carriage *q*. Curves in (B) were computed with the probability of acquiring LA-MRSA from contact with pigs *δ* = *δ*_0_.

Considering now that a farm worker has persistently acquired LA-MRSA and all parameters remained fixed to their baseline value, we explore the probability of occurrence and the duration at which LA-MRSA remained in circulation in the metapopulation (Figure 4A). Results were generally similar to those recorded for *R*_0_, with LA-MRSA carriage failing to persist in the metapopulation if *q*≤0.1 while remaining endemic otherwise. When *q* = 0.05, 26 weeks were required for LA-MRSA carriage to be eliminated from the metapopulation, while this would take 47 weeks when *q* = 0.1 (Figure 4A). Note that, despite seeding simulations with a persistent carrier, only 27.5% (95% C.I. 25.1–30.0) and 33.7% (95% C.I. 31.2–36.3) of the iterations showed MRSA ST398 circulating within the first week when *q* = 0.05 and *q* = 0.10, respectively. This is significantly lower than for the two other scenarios where 41.8% (95% C.I. 39.1–44.5) and 48.1% (95% C.I. 45.4–50.8) of iterations showed presence of carriage when *q* = 0.2 and *q* = 0.35, respectively.

When regular exposure to positive pigs occurs, Figure 4B indicates that the repeated acquisition of MRSA ST398 by exposed populations drastically increases the likelihood of persistence in the metapopulation, with a weekly probability fluctuating around 0.8 and 1 after 3 weeks (Figure 4B).

### Role of Persistent Exposures to Pigs

**Figure 5:**
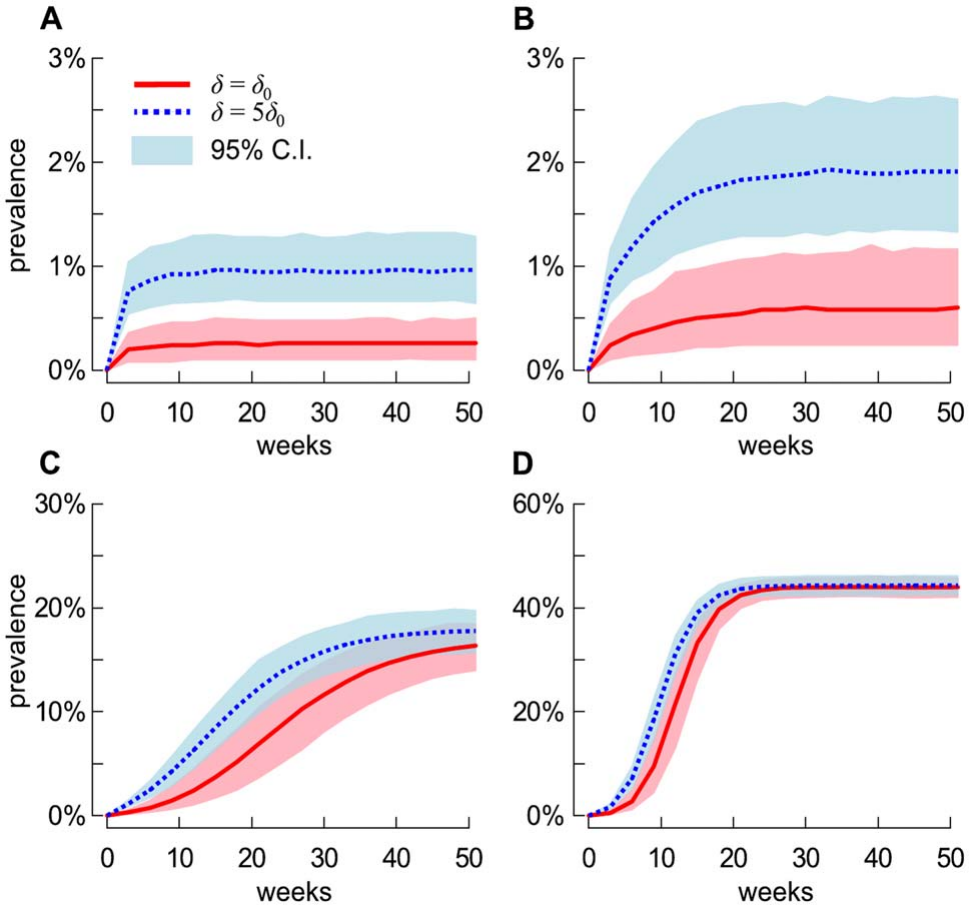
Temporal evolution of the prevalence of MRSA ST398 carriage in humans. Temporal evolution of the prevalence of LA-MRSA carriage in humans when recurrent contacts with pigs occur. Curves were computed for each considered scenario of the probability of persistent carriage *q* and probability acquisition from pigs *δ*: (A) *q* = 0.05, (B) *q* = 0.10, (C) *q* = 0.20 and (D) *q* = 0.35.

Figures 5A to 5D show the mean prevalence of LA-MRSA carriers in humans through time for one year of regular exposure to positive pigs and for each considered scenario of the probability of persistent carriage *q*. Clearly, higher *q* values yielded higher endemic prevalence in humans. After one year, our model suggests that 14% to 20% and 42% to 46% of the human population involved in the metapopulation would carry LA-MRSA if *q* = 0.2 and 0.35, respectively. This is significantly greater than when *q*≤0.1, for which prevalence does not exceed 3 per cent.

Previously, we assumed that biosecurity measures were carried out to stop contacts occurring between FCA and pigs. Departing from this assumption by allowing contacts yielded, however, no significant difference in human prevalence (P>0.85), with the temporal variation of prevalence of LA-MRSA carriage closely matching the dynamics shown in Figure 5 (data not shown).

As an indication of varying exposure to LA-MRSA, we studied the effect of two values of the probability of acquiring MRSA ST398 due to contacts with pigs: *δ* = *δ*_0_ and *5δ*_0_. Increasing *δ* significantly influenced the level of endemic prevalence only in the situation where *q*≤0.1, otherwise little effect was observed (Figure 5). Also, varying *δ* did not affect the role of pig-exposed populations on LA-MRSA transmission dynamics, with risk measures similar for *δ* = *δ*_0_ and 5*δ*_0_. Thus, we presented only the outcomes for *δ* = *δ*_0_, but provided results for *δ* = 5*δ*_0_ in the supporting Figure S1.

**Figure 6:**
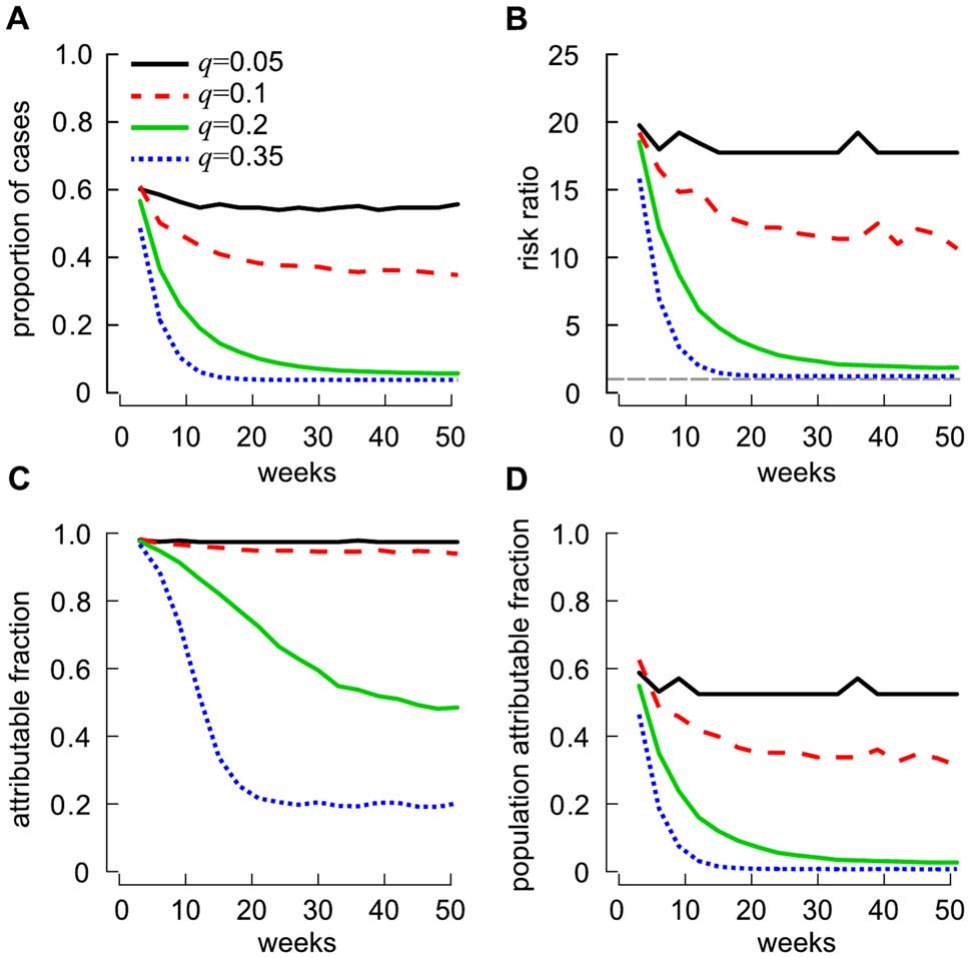
Role of exposure to pigs on the evolution of MRSA ST398 carriage, *δ* = *δ*_0_. Role of exposure to pigs on the evolution of MRSA ST398 carriage when LA-MRSA is recurrently acquired of from pigs with a probability *δ* = *δ*_0_ (similar for *δ = *5*δ*_0_, see Figure S1). (A) The proportion of exposed carriers among all carriers. (B) Ratio of the incidence risk of LA-MRSA carriage in pig-exposed individuals with that in individuals with no direct contact with pigs. (C) The proportion of LA-MRSA carriage in the exposed populations attributable to exposure with pigs (i.e. the attributable fraction). (D) The proportion of LA-MRSA carriage in the population that is attributable to exposure to pigs (i.e. the population attributable fraction). The horizon dashed grey lines in (B) represents the risk ratio = 1.

When *δ* = *δ*_0_ and the probability of persistent carriage was low (*q* = 0.05), individuals that had regular exposure to pigs accounted for 55.5% (95% C.I. 26.9–85.7) of the total number of LA-MRSA carriers recorded after one year (Figure 6A). This represented an incidence risk of LA-MRSA carriage 16.4 times (95% credible interval (Cr.I.) 5.48–32.9) greater than the incidence risk in those that had no exposure (Figure 6B). However, increasing *q* induced a marked reduction in the risk of LA-MRSA carriage due to regular exposure to pigs. After one year, the risk of LA-MRSA carriage in individuals with direct contact to pigs was found to be 9.4 (95% Cr.I. 2.99–19.4), 1.74 (95% Cr.I. 1.17–2.35) and 1.19 (95% Cr.I. 0.98–1.39) times greater than the risk of LA-MRSA carriage in populations with no direct exposure to pigs when *q* = 0.1, 0.2, or 0.35, respectively (Figure 6B). If *q* = 0.35, pig-exposed individuals would represent <6% of the total number of carriers present in the metapopulation (Figure 6A).

We also studied the proportion of pig-to-human and human-to-human transmission mechanisms when LA-MRSA reached endemic states for both pig-exposed individuals and the whole metapopulation for the four considered scenarios (Figures 6C, D). As expected, our model suggests that, in the exposed population: (1) LA-MRSA carriage was mostly (>90%) attributable to exposures to pigs when the probability of persistent carriage is low (*q*≤0.1); and (2) increasing *q* would increase the importance of human-to-human transmission (Figure 6C). While a similar pattern can be observed for the whole population, smaller proportions of LA-MRSA carriage could be attributable to exposures to pigs (Figure 6D). This finding indicates that human-to-human transmission is still an important feature in the transmission dynamics of MRSA ST398 in the community. In particular, Figure 6D indicates that nearly 40% of LA-MRSA carriers were not attributable to exposures to pig when low (*q* = 0.05) probability of persistent carriage is considered.

**Figure 7:**
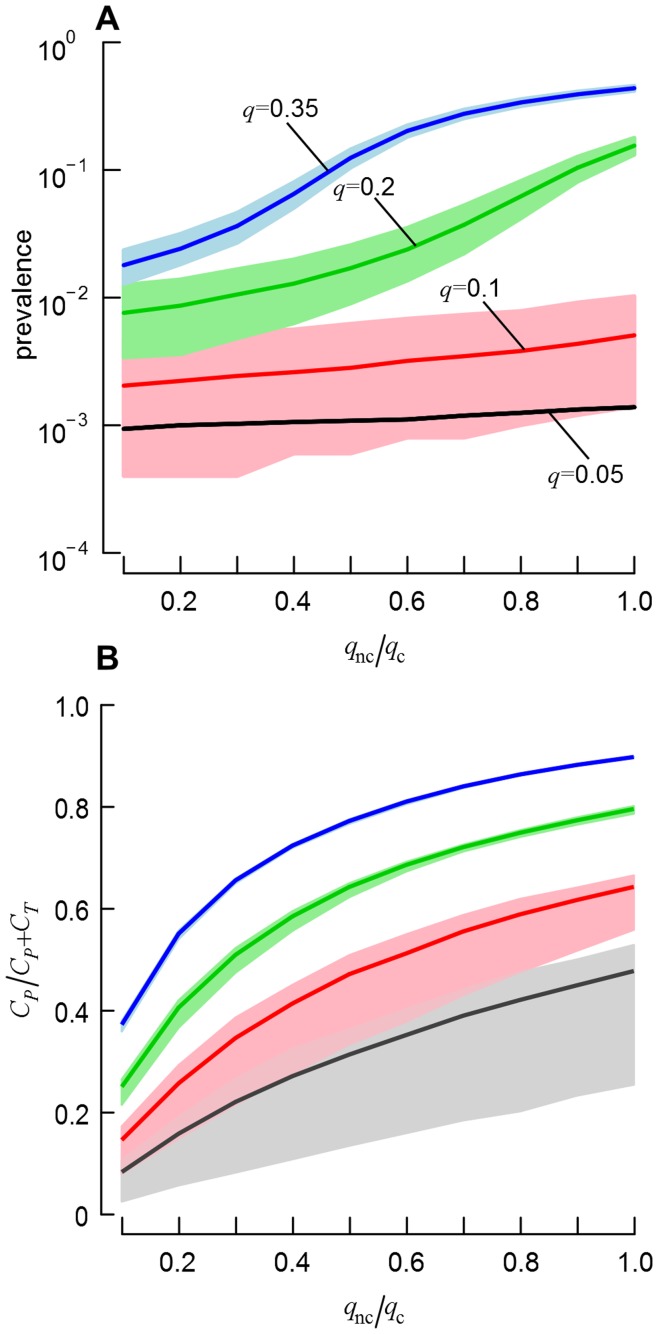
Influence of *q_nc_*/*q_c_* on MRSA ST398 carriage. (A) Evolution of the endemic prevalence when varying the probability of persistent carriage for individuals that show no contacts with live pigs, *q_nc_*, while fixing the probability of persistent carriage in individuals that show contacts with livestock *q_c_*, such as *q_c_* = *q*. (B) Evolution of the proportion of carriers that are persistent among the unexposed populations as a function of *q_nc_*/*q_c_*. Note that, for clarity, no confidence intervals for the scenario *q* = 0.05 were plotted in (A).

It has been shown that persistence of MRSA ST398 in humans is dependent on intensity of animal contact [23], suggesting that LA-MRSA is mostly transient in most humans. Let now discriminate the probability of persistent carriage in those that show contacts with livestock *q_c_* and those that do not, *q_nc_*. We can then explore the behaviour of the model when the effect of pig exposure on the probability of persistent carriage is considered, fixing *q_c_ = q* and increasing *q_nc_* from 0.1*q_c_* to *q_c_*. Figure 7A shows the changes in endemic prevalence when varying the ratio *q_nc/_q_c_* for the four considered scenarios of *q*. Unlike values recorded when *q*≤0.1, decreasing the probability of persistent carriage for unexposed individuals significantly reduces the overall prevalence when *q*≥0.2. Regarding the changes in the within-group prevalence estimates, changes in values were significant when *q* = 0.35, with a 56-times and 60-times reductions in GH and V within-group prevalence, respectively, when *q_nc/_q_c = _*0.1 (Figure S2). Note, however, that only non-null mean within-group prevalence values were recorded (Figure S2), and that some persistent carriers would still remain in the unexposed populations (Figure 7B). These findings highlight the facts that contamination events between exposed and unexposed can still occur, and that chain of infections may remain within unexposed populations, even if the probability of persistent carriage in those that do not show livestock exposure is reduced drastically.

## Discussion

In this study, we developed a metapopulation model to investigate the capacity of MRSA ST398 to spread into and persist in a human population and identify which determinants are most influential in its transmission dynamics. This model was primarily inspired by the need for reliable epidemiological information regarding MRSA ST398 in order to provide guidelines for the implementation of control policies in a country where MRSA ST398 has not yet been reported in pigs, such as the UK. The reports of a new variant of LA-MRSA in cattle in the UK [32] and the widespread distribution of MRSA ST398 in both livestock and humans in Europe [9], [33] has rendered the need for modelling LA-MRSA transmission in humans even more important. Assessing how an emerging LA-MRSA strain may spread from livestock to the community is nonetheless complex as information on the transmissibility of LA-MRSA from livestock to humans is scarce. Furthermore, even though ample evidence of the occupational risks due to physical contacts with livestock have so far been provided [2], [22], [34], [35], the underlying mechanisms are still not well understood.

We showed that introducing MRSA ST398 in a totally susceptible human population may result in an outbreak (i.e. *R*_0_>1) if a relatively large proportion of persistent carriers occurs. For instance, when 20% to 35% of carriers are persistent, the model indicates that prevalence of LA-MRSA in humans quickly rises to reach 17% to 44.5%. While these figures may look alarming, they should be interpreted with caution as recent studies looking at the proportion of farmers in contact with livestock in the Netherlands have indicated that carriage events are most often transient, with a proportion of persistent carriers around 7% [23]. If these findings are externally valid, this would imply that our scenarios with *q*≤0.10 are more realistic to describe the MRSA ST398 transmission dynamics. In these situations, MRSA ST398 is unlikely to get established in the metapopulation (i.e. *R*_0_<1) and would be eliminated relatively quickly after a single incursion (Figure 4A). As expected, being exposed to pigs is an important risk factor for acquiring MRSA ST398, yielding an incidence risk 16.4 (*q* = 0.05) and 9.4 (*q* = 0.10) times greater than when no exposure is present (Figure 6B). This is directly comparable to previously reported results indicating that carriers were 12 times more likely to be pig farmers than non-farmers [34]. However, when exploring the effect of increasing probability of acquiring LA-MRSA (*δ*) for those that are in contact with pigs, different patterns emerge. Despite increasing the value of *δ* from *δ*_0_ to 5*δ*_0_, the incidence risk did not significantly vary, with <20% difference between these two scenarios. In contrast, the prevalence in humans rose, increasing by 6–16 times when *q*≤0.10. These observations suggest that it is the repeated acquisition of LA-MRSA from livestock (i.e. 1/*Γ*_i_), independently of being transient or persistent, rather than the level of exposure (i.e. *δ*), that most influences the epidemiology of LA-MRSA in humans. However, this does not preclude the fact that increasing *δ* would increase the level of endemic prevalence in humans, probably by extending the duration of LA-MRSA carriage of those that are exposed.

It is worth noting that the probability of acquiring LA-MRSA from livestock (*δ*) is not only related to the duration exposure to positive animals and contaminated dust [5], [22], but also directly related to animal prevalence [5]. This would render possible the evaluation of the impact of pig prevalence on that of humans. However, this relation is complex as it relies on a number of properties and characteristics of (*i*) the MRSA strain, (*ii*) the host species, and (*iii*) the husbandry practices. Better defining *δ* may then provide a basis for further refinement and exploitation of our model.

For simplicity, our model ignored transmission of MRSA ST398 between individuals through the environment. While this would have been an important limitation in the context of hospital and community acquired MRSA [36], investigations amongst workers in Dutch pig slaughterhouses workers suggest that transmission of MRSA ST398 between individuals through environmental contamination is a minor transmission route [37], [38]. Subject to the validity of these observations to all at-risk populations involved in our metapopulation, the absence of this route in our model is unlikely to change drastically our inferences.

The metapopulation framework is a standard approach in both human and animal epidemiology to capture the heterogeneity in contacts between at-risk populations [39]. However, the absence of data describing the complex contact structure between the different populations rendered difficult the analysis of MRSA ST398 transmission dynamics from farm to the community. Until empirical data are available to properly parameterise our contact matrix, inference from our results should be done with caution. However, evidences suggest that our outcomes did not suffer from such uncertainty. First, the sensitivity analysis showed the strong resilience of *R*_0_ to variations in contact parameters (Table 2, Figure 3), which requires significant efforts reducing contact pattern to alter the epidemiological behaviour of LA-MRSA in human. Second, we obtained a good fit with observed trends when exploring endemic prevalence levels of MRSA ST398 in each at-risk population. When *q* = 0.05 and *δ = δ*_0_ and 5*δ*_0_, MRSA ST398 is, on average, carried by 17% to 49% of pig farmers while carriers represent 0.07% to 0.25% of the members of community without contacts with pigs (Table 3). These findings are in-line with data from The Netherlands, reporting that MRSA ST398 is carried by nearly 27% of pig farmers (95% C.I. 16.1–40.4%) while it was found on only 0.19% of individuals without livestock-contact [15]. We believe, therefore, that the results here are robust and that the model framework provides a basis for better understanding the epidemiology of LA-MRSA in human populations.

**Table 3:** Point prevalence, and its range, of MRSA ST398 carriage after one year for all human populations involved in the metapopulation.

		Prevalence (min-Max) of MRSA ST398 carriage (in %)					
q	δ	V	VP	T	SHW	F	GH
0.05	δ_0_	0.09 (0–12.5)	0.39 (0–28.6)	0.59 (0–8.82)	1.52 (0–11.3)	16.9 (0–55.5)	0.07 (0–0.46)
	5δ_0_	0.27 (0–25.0)	2.52 (0–57.1)	2.52 (0–14.7)	5.72 (0–20.6)	49.4 (0–100)	0.24 (0–0.74)
0.1	δ_0_	0.49 (0–18.8)	0.90 (0–28.6)	1.40 (0–11.8)	4.83 (0–24.7)	23.7 (0–100)	0.37 (0–1.30)
	5δ_0_	1.45 (0–25.0)	5.09 (0–42.9)	5.75 (0–23.5)	14.6 (1.03–35.1)	58.9 (11.1–100)	1.07 (0.26–2.19)
0.2	δ_0_	20.9 (0–62.5)	10.4 (0–71.4)	13.0 (0–71.4)	33.4 (14.4–51.5)	38.7 (0–88.9)	14.6 (10.6–18.4)
	5δ_0_	21.9 (0–62.5)	17.6 (0–71.4)	19.7 (0–50.0)	39.7 (17.5–62.9)	70.0 (22.2–100)	15.8 (11.8–19.1)
0.35	δ_0_	52.0 (12.5–93.8)	34.1 (0–100)	36.6 (11.8–64.7)	60.3 (40.2–76.3)	57.8 (0–100)	41.9 (38.6–44.9)
	5δ_0_	51.8 (18.8–87.5)	39.9 (0–100)	41.6 (11.8–73.5)	62.6 (42.3–81.4)	78.9 (44.4–100)	42.1 (39.3–45.0)

Prevalence stratified for each considered scenario of the probability of persistent carriage *q* and probability acquisition from pigs *δ.*

F: workers of the pig farm, VP: pig veterinarians, T: transporters, SHW: slaughterhouse workers, V: small animal veterinarians, GH: general human population.

Because we were primarily interested in the spread from a single contaminated farm to the community, we did not account for the multiple exposures of some populations to livestock. However, recurrently contacting live animals from different farms has the potential to increase the probability of acquiring LA-MRSA. This observation limits our ability to evaluate the importance of pig veterinarians, transporters and slaughterhouse workers in the spread of MRSA ST398 in the community. It is therefore not surprising that prevalence data produced by our model for pig veterinarians (i.e. from 0.39% to 5.09% when *q*≤0.1, Table 3) are 3 to 115 times lower than what has been previously reported (i.e. 12.5% [35] and 45% [40]). Whether these increases in prevalence would increase the risk of MRSA ST398 to spread in the community is unknown. However, it can be hypothesised that they may contribute (at least to some degree) to the spread of LA-MRSA between farms as suspected for other antimicrobial-resistant organisms [41]. This would in-turn affect the course of epidemics in the community.

Simulations showed that, once MRSA ST398 is established in pigs, elimination from the community would be impossible (Figures 4B and 7) and prevalence in humans would be expected to increase until endemicity is reached (Figure 5). Control efforts should therefore focus on reducing the intra-herd prevalence of LA-MRSA in positive farms, and preventing its transfer from pigs to humans. Benefit of such an approach is two-fold. First, the repeated acquisition of bacteria by individuals exposed to pigs would be limited, since the probability of acquiring MRSA ST398 in humans is directly related to the intra-herd prevalence [5]. Second, it would reduce the spread of MRSA ST398 between farms [4]. However, controlling LA-MRSA on the farm is difficult, notably due to logistic and economic constraints as well as the lack of disinfection products licensed. The implementation of preventive measures that limit the transmission of LA-MRSA between humans (i.e. reducing the value of *β* and/or *µ_P_,* Figure 3A) and from livestock to humans (i.e. reducing the value of *δ,* Figure 4) should therefore be considered while cost-effective products against MRSA ST398 in farms are being developed.

In conclusion, the work presented in this study highlights the need for the development of effective prevention, and addresses several public health issues regarding the spread of MRSA ST398. We demonstrated the key role of the probability of persistent carriage in the MRSA ST398 transmission dynamics indicating a need to confirm the frequency of this event in real populations. We further showed that the presence of recurrent exposures with pigs in risky populations allows LA-MRSA to persist in the metapopulation and transmission events to occur beyond the farming community, even when a low probability of persistent carriage is considered. Although prevalence estimates outside the risk populations were predicted low, MRSA ST398 eradication is thus unlikely if its presence remain in livestock. Therefore reinforcing measures at the farm level to reduce MRSA ST398 transmission from livestock to humans should be the cornerstone of control policies for driving its eradication from the community.

## Supporting Information

Figure S1 **Role of exposure to pigs on the evolution of MRSA ST398 carriage,** ***δ*** **= 5** ***δ*** **_0_.** Role of exposure to pigs on the three-weekly evolution of MRSA ST398 carriage when recurrent acquisition of MRSA from pigs is occurring with a probability *δ* = 5*δ*_0_. (A) The proportion of exposed carriers among all carriers. (B) Ratio of the incidence risk of MRSA carriage in individuals with direct contact with pigs compared to individuals with no direct contact with pigs. (C) The proportion of MRSA ST398 carriage in the exposed group attributable to exposure with pigs (i.e. the attributable fraction). (D) The proportion of MRSA carriage in the population that is attributable to exposure to pigs (i.e. the population attributable fraction). The horizon dashed grey lines in (B) represents the risk ratio = 1.
(TIF)

Figure S2 **Influence of** ***q_nc_*** **/** ***q_c_*** **on the endemic MRSA ST398 prevalence in the different human population at-risk.** Line plot showing the changes in endemic prevalence for all human populations involved in the metapopulation when varying the probability of persistent carriage for individuals that show no contacts with live pigs, *q_nc_*, while fixing the probability of persistent carriage in individuals that show contacts with livestock *q_c_*, such as *q_c_* = *q*. Curves were computed for each considered scenario of the probability of persistent carriage *q*: (A) *q* = 0.05, (B) *q* = 0.10, (C) *q* = 0.20 and (D) *q* = 0.35.
(TIF)

Text S1 **Structure of the population and contact distribution.** Further details on the structure of the population and the contact distribution that were considered in the model are presented.
(DOC)
