# Supplementary figures and images for: A Metapopulation Model to Assess the Capacity of Spread of Meticillin-Resistant Staphylococcus aureus ST398 in Humans
Source: PLoS One. 2012 Oct 24;7(10):e47504. doi: 10.1371/journal.pone.0047504 (PMC3480390; doi:10.1371/journal.pone.0047504)

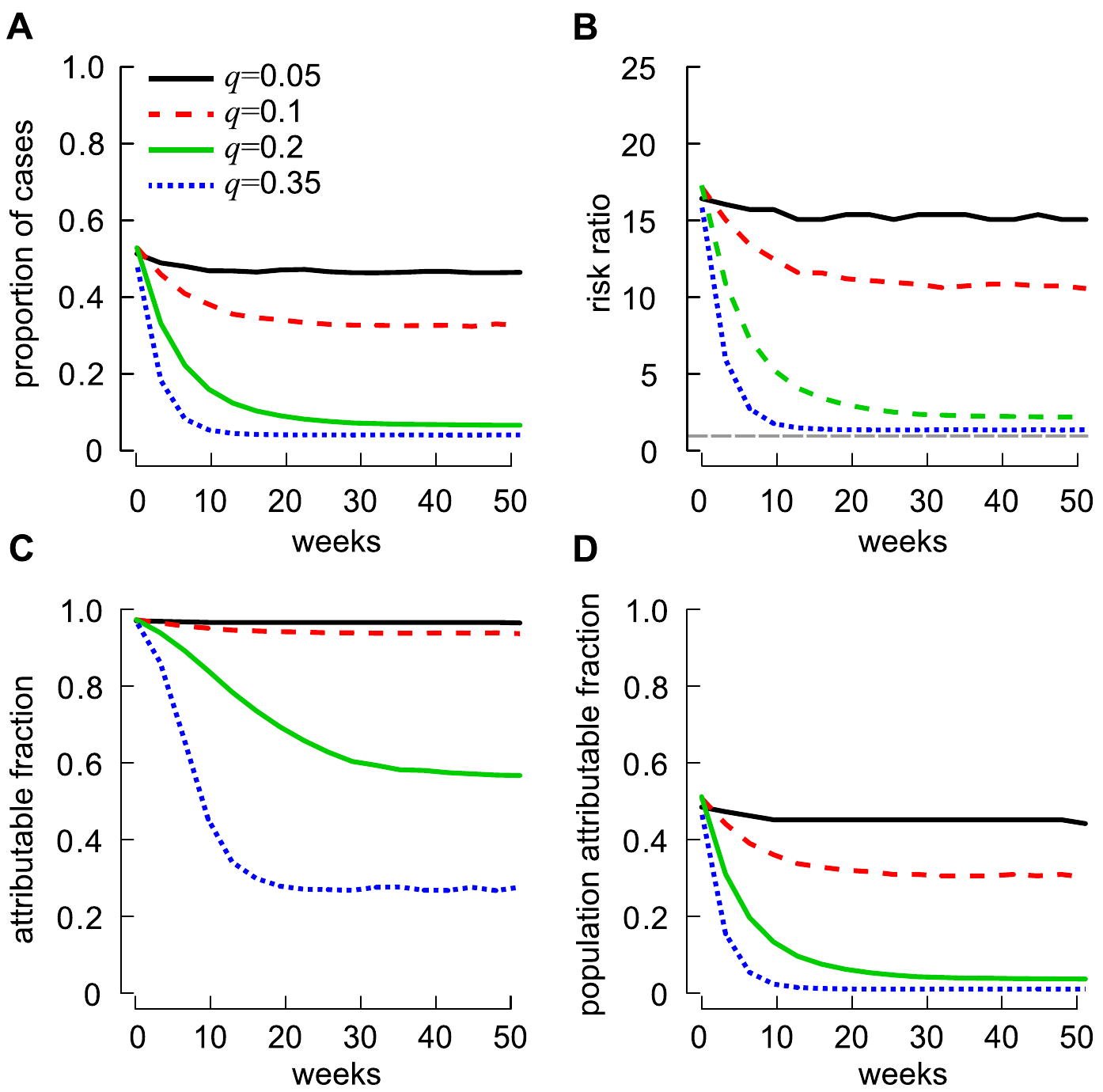

Supplement: Figure S1 — Role of exposure to pigs on the evolution of MRSA ST398 carriage, δ = 5 δ 0. Role of exposure to pigs on the three-weekly evolution of MRSA ST398 carriage when recurrent acquisition of MRSA from pigs is occurring with a probability δ = 5δ 0. (A) The proportion of exposed carriers among all carriers. (B) Ratio of the incidence risk of MRSA carriage in individuals with direct contact with pigs compared to individuals with no direct contact with pigs. (C) The proportion of MRSA ST398 carriage in the exposed group attributable to exposure with pigs (i.e. the attributable fraction). (D) The proportion of MRSA carriage in the population that is attributable to exposure to pigs (i.e. the population attributable fraction). The horizon dashed grey lines in (B) represents the risk ratio = 1. (TIF) [file pone.0047504.s001.tif]

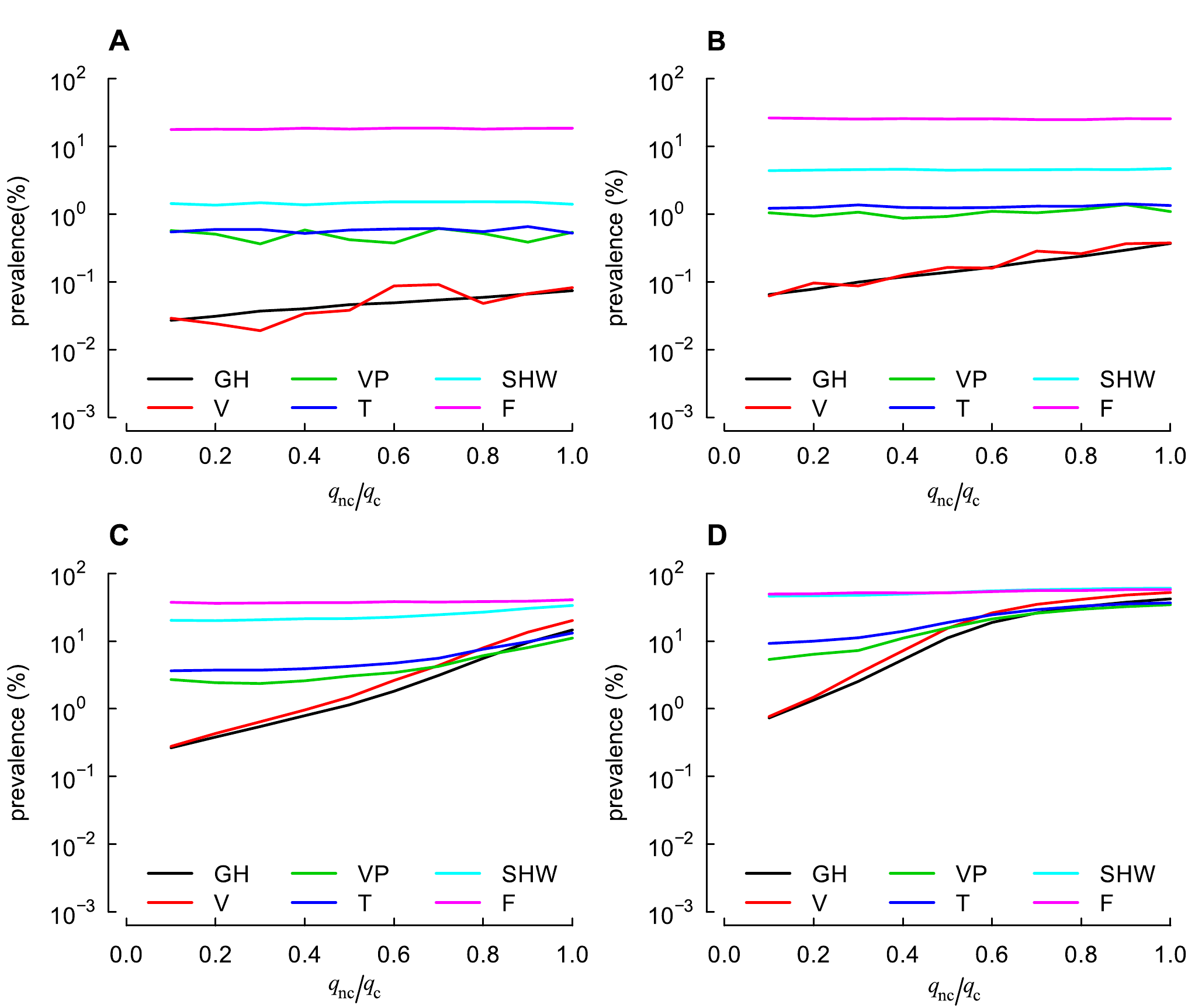

Supplement: Figure S2 — Influence of qnc / qc on the endemic MRSA ST398 prevalence in the different human population at-risk. Line plot showing the changes in endemic prevalence for all human populations involved in the metapopulation when varying the probability of persistent carriage for individuals that show no contacts with live pigs, qnc, while fixing the probability of persistent carriage in individuals that show contacts with livestock qc, such as qc = q. Curves were computed for each considered scenario of the probability of persistent carriage q: (A) q = 0.05, (B) q = 0.10, (C) q = 0.20 and (D) q = 0.35. (TIF) [file pone.0047504.s002.tif]
