# Supplementary material for: A Metapopulation Model to Assess the Capacity of Spread of Meticillin-Resistant Staphylococcus aureus ST398 in Humans
Source: PLoS One. 2012 Oct 24;7(10):e47504. doi: 10.1371/journal.pone.0047504 (PMC3480390; doi:10.1371/journal.pone.0047504)
Supplement: Text S1 — Structure of the population and contact distribution. Further details on the structure of the population and the contact distribution that were considered in the model are presented. (DOC) [file pone.0047504.s003.doc]

**Electronic Supplementary Material, Text S1 PLoS One**

**A metapopulation model to assess the capacity of spread of methicillin-resistant *Staphylococcus aureus* ST398 in humans**

T. Porphyre, E. S. Giotis, D.H. Lloyd, K.D.C. Stärk

Department of Veterinary Clinical Sciences, Royal Veterinary College, Hatfield AL9 7TA, UK

This electronic appendix presents in detail the structure of the population and the contact distribution used to compute and analyse the capacity of spread in a human population of the meticillin-resistant *Staphylococcus aureus* (MRSA) sequence type (ST) 398.

# STRUCTURE OF POPULATION

To illustrate how the model performs, we considered a single pig farm Norfolk, East Anglia, UK, where there is a high density of pigs (June Survey of Agriculture and Horticulture, 2009, [http://www.defra.gov.uk/statistics/foodfarm/landuselivestock/junesurvey/junesurveyresults](http://www.defra.gov.uk/statistics/foodfarm/landuselivestock/junesurvey/junesurveyresults/)). We assumed that at-risk individuals do not acquire MRSA ST398 from more than one source farm. The size of the population at-risk for directly or indirectly acquiring MRSA ST398 from this given pig farm is then equivalent to the density of the human population per farm. Since 170 farms raise pigs for commercial purpose in Norfolk (June Survey of Agriculture and Horticulture, 2009) for an estimated total human population of 847,300 individuals (Subnational Statistics Unit, Office for National Statistics, 2008, <http://www.norfolkinsight.org.uk/dataviews/view?viewId=83>), we considered a total human population at risk of MRSA ST398 infection of 4984 individuals per farm.

Whilst the exact structure of the population at risk is unknown, we extrapolated the demographic structure of the metapopulation from various specific sources. The average number of individuals working in a UK pig farm and in direct contact with live pigs (*n*=3.9) was extracted from a survey of 147 English pig farms .

We also assumed that all pigs produced in a given pig farm are slaughtered in only one slaughterhouse and only one company of transporters served this slaughterhouse. We then restricted the number of at-risk slaughterhouse workers (SHW) to those working in the lairage (where animals are kept before being slaughtered) and dirty area (where animals are slaughtered) as recent survey from The Netherlands showed that only those that are in direct contact with live pigs are more likely to carry MRSA ST398 . Although size of the population working directly with live pigs may vary between slaughterhouses, we considered a slaughterhouse with a similar organisation and population structure as that described in , showing about 41 employees (i.e. 30 in lairage, 10 in dirty area and 1 veterinarian) working in direct contact with live pigs.

The number of transporters (T) involved in the metapopulation was extrapolated directly from the average number of pigs slaughtered annually per slaughterhouse in the UK and the average capacity of a lorry. Data from 2008 shows there are 131 abattoirs in the UK, 16 of which are specialising in pigs . In 2008, 9,427,000 pigs were slaughtered in UK abattoirs . We then considered that a typical lorry of about 51.2 m^2^ over two levels (2 × 10.28m × 2.49m) can carry 250 kg/m^2^ of pig for carcase quality and welfare . Given that the average weight of a live pig to go to slaughter is about 80kg, we estimated that, for each working days (1 / 52 weeks / 5 open days), each slaughterhouse requires an average of 14 transporters (*n*=9427000/16/52/(250×10.280×2.490/80×2)/5).

The number of veterinarians present in our population at risk was extrapolated directly from the reported density of veterinarians per pig farm. As of 31 March 2010, the Register of Veterinary Practice Premises included 4821 premises in the UK (Annual Report of the Royal College of Veterinary Surgeons 2010, <http://www.rcvs.org.uk/publications/rcvs-facts-2010/>). In the same period, 14843 individuals were registered in the UK as home practicing in general veterinary practice or other. As such, we fixed the average number of veterinarians in veterinary practice as 14843/4821 = 3.1 individuals. In Norfolk, 371 premises were reported as of 31 March 2010 (Royal College of Veterinary Surgeons, 2010). Considering that each premise shows an average number of veterinarians similar to the national average, Norfolk was assumed showing a density of 1.35 veterinarians per 1000 inhabitants. As such, over the 4984 individuals that are involved in the metapopulation, 6.7 veterinarians were considered. We also forced in this population one premise comprising only veterinarians specialised in pig population health, here termed pig veterinarians (VP). Therefore, 3.1 pig veterinarians were included in our study population.

Together with the average household size *h* in the UK (*h*=2.4, Labour Force Survey, Office for National Statistics 2011, [http://www.ons.gov.uk/ons/rel/family-demography/families-and-households/2011/stb-families-households.html#tab-Household-size](http://www.ons.gov.uk/ons/rel/family-demography/families-and-households/2011/stb-families-households.html#tab-Household-size)), we considered a population in the farm subpopulation comprised of 9 individuals. Similarly, SHW, T, V and VP subpopulations of the metapopulation accounted for 97, 34, 16 and 7 individuals in the metapopulation, respectively.

While number of companion animals involved in the metapopulation follows national trends, with 0.75 dogs and cats per household , the number of companion animals present in the pig farm of the metapopulation was extrapolated from the average number of dogs and cats present in UK pig farms (n=3; ). Overall, the metapopulation includes 4984 humans; among which 147 have regular direct and indirect contacts with live pigs, and 1588 companion animals (i.e. dogs and cats).

# CONTACT STRUCTURE

In the absence of any empirical data measuring amount of contact within and between members of each population in Norfolk, we considered that the number contacts *p_i,j_* is the product of three processes generating contact: contacts during work activities (A*_i,j_*), contacts between household members (B*_i_*) and contacts during other daily activities (C*_i,j_*, e.g. leisure, shopping). Based on this assumption, the contact matrix *P_i,j_* was built such that the diagonal provides the number of contacts within each population as the sum of these three processes, whereas household contacts B*_i_* were excluded for all remaining arrays. Note that values of A*_i,j_* were computed as a function of the *j*^th^ population size *N_j_*, the proportions 1/*h* of active individuals (i.e. those that actively participate in the network of contacts between populations, not household members) and the frequency 1/*Γ_i_* at which each population have exposure to live pigs.

For all human populations, values of B*_i_* were defined such as:

$\text{B}_{i}=h-1$ (A.1)

while values of C*_i,j_* were defined such as:

$\text{C}_{i,j}=\frac{N_{j}}{\sum_{n} N_{n}}\prod_{k\neq j} \left(1-\frac{N_{k}}{\sum_{n} N_{n}}\right) \left(1-w-f\right) c$ with *i*, *j*, *n* ∈{F, V, VP, T, SHW, GH} (A.2)

where *c* is the average number of physical and close contacts per day between people in the UK;  *w* is the proportion of contacts that emerge from work; *f* the proportion of contacts that occur at their household; and $\sum_{n} N_{n}$the total number of humans in the metapopulation. Estimates of *c*, *w* and *f* were all derived from the UK subset of the POLYMOD study , a cross-sectional population-based survey of individuals in eight European countries in the year 2005-2006. POLYMOD reported that on average, people in the UK have *c*=11.74 physical and close contacts per day, for which nearly 50% occur outside work and home activities. We assumed that the proportion of contacts that emerge from work or at their household are both constant for each population.

Details on the calculation of the average number of contacts made by members of each human population during their normal work activities, A*_i,j_*; and the calculation of the average number of contacts *P_i,j_* made by companion animal population (i.e. CA and FCA) are given below. Exact values of the contact matrix are given in Table S1.

## Contact between human populations

### Non-exposed human populations

This section presents in detail the average number of contacts *P_i,j_* assumedly made by individuals of each non-exposed human population (i.e. V and GH) with the other populations of the metapopulation during normal daily activities.

*Small animal vets (V) contacts*: Let consider again that *P_i,j_* = A*_i,j_* + B*_i_* + C*_i,j_* when *j* = *i*, and *P_i,j_*= A*_i,j_* + C*_i,j_* otherwise. In the case of individuals that belong to the V population, the number of contacts with other humans from other populations was considered driven by the average number of consultations that a small veterinarian may have in a given day. Given that there are 4821 veterinary clinics in the UK (RCVS 2010) and that a maximum of 30 million consultations are carried out by veterinary surgeons each year (*VR*, October 29, 2009, vol 165, pp 486-487), and assuming that practices are open throughout the year from Monday to Friday, except for vacations (~1month), small animal veterinary practitioners were set to contact an average of *v*=8.4 customers per day. As such, the average number of contacts made by each member of the population V with members of the other human population during working activities was defined as:

$\text{A}_{i,j}=\frac{1}{h}\frac{N_{j}}{\sum_{n} N_{n}} v$, with *i*=V, and *n* ∈{F, V, VP, T, SHW, GH} (A.3)

with *h* the average household size reported in the UK (see above for more details).

In contrast, the number of contacts between small animal veterinarians and individuals of the F population was computed such as all households in the F population own a companion animal and are regular costumers of a single small animal veterinary practice where they contact only one small animal veterinary practitioner at each visit.

We also assumed that pig veterinarians may treat their own companion animals by themselves. In this case, we set A*_i,j_* = 0 when *j*= VP.

The number of contacts A*_i,i_* that occurs between members of the V population is driven by the average size of a veterinary practice (see above for details). Here we arbitrarily considered that surgeons of a veterinary practice will contact each other every day and that their family would meet at least once a week.

*General population (GH) contacts*: The average number of daily contacts *P_i,j_* that members of the population GH make was directly extracted from the POLYMOD data. Because this data informed on the average number of physical contact between individuals from different daily activities, including work, family and leisure, we considered that the number of contacts occurring within the GH population *P_i,i_* was directly extracted from *c* and modified the expression A.2 such as:

$P_{i,i}=\prod_{k\neq i} \left(1-\frac{N_{k}}{\sum_{n} N_{n}}\right) c$ , with *i* = GH and *n* ∈{F, V, VP, T, SHW, GH} (A.4)

while the amount of contacts made by members of the GH population with those of the remaining human populations *j*, *P_i,j_*, was extrapolated such as:

$P_{i,j}=\text{A}_{i,j}+\text{C}_{i,j}=\text{A}'_{i,j}+\frac{N_{j}}{\sum_{n} N_{n}}\prod_{k\neq j} \left(1-\frac{N_{k}}{\sum_{n} N_{n}}\right) c$, with *i* = GH. (A.5)

Because some members of the GH population own at least 1 companion animal, we needed to account for the extra number of contact A’*_i,j_* due to the visit to the small animal veterinarian for the annual health check-up of their animals. In this case, the component A’*_i,j_* of the expression A.5 was added under the assumption that only one person of the household would come with the animal to the veterinarian. Here, we also considered that pets go to see a veterinarian only once a year for vaccine boosters and annual health check-up. Assuming that no extra-contacts occurs with other populations during work activities, we fixed A’*_i,j_*=0 in the expression A.5 for all remaining population *j*.

### Pig-exposed populations

This section presents in detail the average number of contacts *P_i,j_* assumedly made by individuals of each exposed population (i.e. VP, T, SHW and F) with the remaining populations during normal daily activities.

*Pig veterinarians (VP), Transporters (T) and Slaughterhouse workers (SHW)*: Because the relative isolation of populations along the production chain, with a directed flow of pigs going from one element of the chain to another, only T and VP were set to have contact due to working activities A*_i,j_* with individuals involved in the F population. We also considered that no contacts were set to occur between individuals of the T and VP populations, between individuals of the VP and SHW populations, and between individuals of the SHW and F populations except due to normal daily activities (C*_i,j_*).

We also assumed that only 1 transporter is in charge of driving and loading the lorry from farm to slaughterhouse that occurs once every *Γ_i_* = 21 days (3 weeks). We considered that transporters would contact all farm employees and all people working at the lairage for loading and unloading their lorry. Similarly, we considered that veterinarians of the VP population would contact all farm employees during their quarterly (*Γ_i_* =90 days) visit to check the population health of the pig farm. We also assumed that only one veterinarian would come to the farm.

The number of contacts A*_i,i_* that occurs between members of the VP population is driven by the size of the veterinary practice. Similarly to the calculation of the number of contacts occurring within the V population, we arbitrarily considered that pig veterinarians belonging to the same veterinary practice would contact each other every day and that their family would meet at least once a week.

The number of contacts A*_i,i_* that occurs between members of the SHW population depends on the number of workers involved in the two zones of the abattoir where live pigs are present: the lairage (where animals are kept before being slaughtered) and the dirty area (where animals are slaughtered). Following biosecurity measures, we further assumed that individuals belonging to one of these two areas would contact only those working in the same area of the abattoir and that no physical contacts would occur between individuals belonging to the other considered area of the abattoir. Then, the number of contacts occurring between individuals of the SHW population was fixed equal to the arithmetic mean of the number of contacts occurring between individuals working in lairage and dirty area.

Because transporters spend most of their daily working activities isolated in their truck, we assumed that contacts A*_i,i_* that occurs between members of the T population is limited. We therefore assumed that the number of contacts that each members of the T population make is similar to the average number of contact that were reported by individuals involved in the UK subset of the POLYMOD study .

Finally, the number of contacts A*_i,j_* of individuals of the T and SHW populations with those of the V population was similarly calculated to that with those of the non-exposed populations. The number of contacts between members of the T and SHW with small animal veterinarians is related to both the average number of companion animals per household and the frequency that companion animal owners visit veterinarians for health check-up. As seen above, no contact was considered occurring between members of VP and V populations, except for normal daily activities, under the assumption that pig veterinarians were qualified to treat their own companion animal if required.

*Farmers (F) contacts*: Most of the physical contacts that a farm worker makes would be likely to be restricted to contacts with other farm employees, the transporters that drive the farm production to the slaughterhouse, and the veterinarians who come to check the population health of the farm and those who are consulted for the health of the farm companion animals. We therefore assumed that farmers show no physical contacts during working activities with abattoir employees and individuals of the general population. As such, we set A*_i,j_* = 0 when *i*=F and *j*∈{SHW, GH}.

Similarly to our previous assumption, all farm employees would be in contact with all individuals of the VP and T population that come to the farm. We also assumed that only one veterinarian or transporter would come to the farm. Let us now define 1/*Γ_i_* the rate at which veterinarians specialised in pig population medicine (*Γ_i_* = 90 days) or transporters (*Γ_i_* = 21 days) come to the farm, the number of physical contacts that farm employees (but not their family) make with these individuals may be calculated such as:

$\text{A}_{i,j}=\frac{1}{h} \frac{1}{\Gamma_{i}\text{ }}$, with *i*=F, and *j* ∈{VP, T} (A.6)

The number of contacts A*_i,j_* between individuals of the F population with those of the V population was calculated assuming that a single farm worker is going to consult a small animal veterinarian (rather than veterinarian specialised in pig health) for checking the health of the companion animal present on the farm (FCA). We also considered that each farm companion animal would visit the small animal veterinarian independently to the other companion animals present on the farm and at a frequency similar that other companion animals involved in the metapopulation (i.e. once per year).

To calculate the amount of physical contact that occurs during work activities between individuals of the F population, A*_i,i_*, we made the distinction between the farm manager and farm employees. While the former lives on farm with his family, the latter live outside the farm with his family. As such, we considered that the number of physical contacts A*_i,i_*, is the average number of physical contacts that occurs between farm employees, the manager and the manager’s family. To do so, we assumed that farm employees would contact every day all workers present on farm as well as all members of the family of the farm manager. On the other hand, the farm manager and his family were assumed to contact every day all employees but not their family.

## Contacts made by companion animal populations

This section presents in detail the average number of daily contacts *P_i,j_* assumedly made by individuals of each companion animal population (i.e. CA and FCA) with the other populations of the metapopulation.

To calculate the number of contacts that companion animals may have, we based our analysis on the study of Westgarth *et al*. investigating the nature and frequency of the contacts that occur between dogs, and between dogs and people by means of a questionnaire survey. In this study, 260 dog-owning households in a community in Cheshire, UK, participated in the study by answering questions related to their behaviour, and notably the numbers of people and dogs that met and interacted daily during the week and at weekends during normal daily walks. Data showed that most of the dogs were estimated by their owners to meet and interact with one to five other dogs per day outside the household, with one to two being most common for weekdays and three to five at weekends. Here we considered that dogs contact an average of 3 other dogs per day outside the household. We added the number of contacts dogs may make with other companion animals (dogs and cats) inside the household, such as it is equal to the average number of companion animals in pet owing households minus one. Given that in 2010, 13 million household have at least one pet in the UK (PFMA, Pet Population 2010, www.pfma.org.uk/statistics/index.cfm?cat_id=60 [accessed January 2011]) with around 20.8 million dogs and cats , the number of companion animals in pet owing households averaged around 1.6 animals. Given the paucity of data related to the number of contacts between cats, we also assumed that cats would show a similar number of daily contacts as dogs.

Contacts with humans were made in 2 ways: contacts during regular walks (dog) or roaming (cat); and during their annual visit to the small animal veterinarian.

First of all, let consider that Norfolk shows similar population structures of cats and dogs as the national figures, showing 50.5% dogs and 49.5% cats . The average number of contacts that companion animals (dogs and cats) may have with people can be extrapolated such as *P_i,j_* = 0.505 × *P_i,j_*(dogs) + 0.495 × *P_i,j_*(cats). Given , most of the dogs were estimated by their owners to meet and interact with three to five people per day outside their household. Here we considered that dogs contact on average 4 people outside their household plus those present within their household, *P_i,j_*(dogs) = 4 + *h*. We extrapolated the number of people that cats may contacts *P_i,j_*(cats) by computing the number of households cats may visit during their daily roaming.

Using radio-telemetry and collar-mounted activity sensors to evaluate the home range size of owned and unowned free-roaming cats in Illinois, USA, Horn *et al*. found that owned cats show an annual home range averaging around 2.82 hectares, equivalent to that found in Canberra, Australia . Therefore, given that Norfolk shows, on average, 1.5 households per hectare (537100/(847300/2.36)) we extrapolated the number of households domestic cats may visit such as 2.82 × 1.5=4.2. Together with the average number of people in an household *h*, the number of people that cats may contact per day is *P_i,j_*(cats) = 4.2 × *h* = 9.91.

Second, all companion animals involved in the metapopulation were set to annually go to visit the small animal veterinarian for vaccine boosters and annual health check.

**Table S1.** Contact matrix *P_i,j_* showing the average number of contacts made by each member of the population *i* with those of the population *j* during their normal daily activities. Matrix is read such as: in each day, members of the population *i* contact *p_i,j_* members of the population *j*. For example, in any given day we assumed that pig vets VP will contact about 5.688 members of the general population (GH) while members of the GH population would contact on average 0.017 pig veterinarians.

| **Population** | **Population *i*** | | | | | | | |
| --- | --- | --- | --- | --- | --- | --- | --- | --- |
| ***j*** | **F** | **FCA** | **T** | **SHW** | **V** | **CA** | **GH** | **VP** |
| **F** | 3.475 | 5.318 | 0.013 | 0.011 | 0.011 | 0 | 0.021 | 0.017 |
| **FCA** | 1.767 | 2.103 | 0 | 0 | <0.001 | <0.001 | 0 | 0.005 |
| **T** | 0.060 | 0 | 2.373 | 4.700 | 0.064 | 0.054 | 0.079 | 0.039 |
| **SHW** | 0.113 | 0 | 0.433 | 12.01 | 0.182 | 0.156 | 0.225 | 0.113 |
| **V** | 0.012 | 0.028 | 0.022 | 0.012 | 4.150 | 0.028 | 0.022 | 0.010 |
| **CA** | 0 | 3.600 | 0.752 | 0.015 | 4.320 | 3.600 | 0.752 | 0 |
| **GH** | 5.688 | 0 | 5.688 | 5.688 | 9.162 | 7.867 | 11.37 | 5.688 |
| **VP** | 0.013 | 0.011 | 0.008 | 0.008 | 0.008 | 0 | 0.017 | 2.950 |

F: workers of the pig farm, VP: pig veterinarians, T: transporters, SHW: slaughterhouse workers in dirty zone, V: veterinarians, GH: general human population

# REFERENCES

1. Alarcon P, Velasova M, Mastin A, Nevel A, Stärk KDC, et al. (2011) Farm level risk factors associated with severity of post-weaning multi-systemic wasting syndrome. Prev Vet Med 101: 182-191. DOI: 10.1016/j.prevetmed.2011.06.001.

2. Alarcon P, Velasova M, Werling D, Stärk KDC, Chang Y-M, et al. (2011) Assessment and quantification of post-weaning multi-systemic wasting syndrome severity at farm level. Prev Vet Med 98: 19-28. DOI: 10.1016/j.prevetmed.2010.09.022.

3. van Cleef B, Broens EM, Voss A, Huijsdens X, Züchner L, et al. (2010) High prevalence of nasal MRSA carriage in slaughterhouse workers in contact with live pigs in The Netherlands. Epidemiol Infect 138: 756-763.

4. BPEX (2009) Pig yearbook 2009. Milton Keynes, UK: British Pig Executive.

5. Guise HJ, Penny HC (1989) Factors influencing the welfare and carcass and meat quality of pigs. 1. The effects of stocking density in transport and the use of electric goads. Anim Prod 49: 511-515.

6. Warriss P (1998) Choosing appropriate space allowances for slaughter pigs transported by road: a review. Vet Rec 142: 449-454.

7. Murray JK, Browne WJ, Roberts MA, Whitmarsh A, Gruffydd-Jones TJ (2010) Number and ownership profiles of cats and dogs in the UK. Vet Rec 166: 163-168. DOI: 10.1136/vr.b4712.

8. Mossong J, Hens N, Jit M, Beutels P, Auranen K, et al. (2008) Social contacts and mixing patterns relevant to the spread of infectious diseases. PLoS Med 5: e74.

9. Westgarth C, Pinchbeck GL, Bradshaw JWS, Dawson S, Gaskell RM, et al. (2008) Dog-human and dog-dog interactions of 260 dog-owning households in a community in Cheshire. Vet Rec 162: 436-442. DOI: 10.1136/vr.162.14.436.

10. Horn JA, Mateus-Pinilla N, Warner RE, Heske EJ (2011) Home range, habitat use, and activity patterns of free-roaming domestic cats. J Wildl Manage 75: 1177-1185. DOI: 10.1002/jwmg.145.

11. Barratt DG (1997) Home range size, habitat utilisation and movement patterns of suburban and farm cats *Felis catus*. Ecography 20: 271-280. DOI: 10.1111/j.1600-0587.1997.tb00371.x.
